# Analysis of the Breast Cancer Journey in Namibia

**DOI:** 10.1001/jamanetworkopen.2023.41402

**Published:** 2023-11-03

**Authors:** Pauline Boucheron, Annelle Zietsman, Johanna Pontac, Rolf Hansen, Benjamin O. Anderson, Kayo Togawa, Peter M. Macharia, Milena Foerster, Joachim Schüz, Isabel dos-Santos-Silva, Valerie McCormack

**Affiliations:** 1 International Agency for Research on Cancer, Environment and Lifestyle Epidemiology Branch, Lyon, France; 2 AB May Cancer Centre, Windhoek Central Hospital, Windhoek, Namibia; 3 Cancer Association of Namibia, Windhoek, Namibia; 4 University of Washington, Seattle; 5 World Health Organization, Geneva, Switzerland; 6 National Cancer Centre Institute for Cancer Control, Division of Population Data Science, Tokyo, Japan; 7 Population Health Unit, Kenya Medical Research Institute–Wellcome Trust Research Programme, Nairobi, Kenya; 8 Centre for Health Informatics, Computing, and Statistics, Lancaster Medical School, Lancaster University, Lancaster, United Kingdom; 9 Department of Public Health, Institute of Tropical Medicine, Antwerp, Belgium; 10 Department of Non-Communicable Disease Epidemiology, London School of Hygiene and Tropical Medicine, London, United Kingdom

## Abstract

**Importance:**

Breast cancer (BC) is the leading cancer among women in Namibia. Examining the BC journey in this multiracial country where inequalities remain large is needed to inform effective interventions to reduce BC mortality.

**Objective:**

To describe the entire BC journey of Namibian women by race, utilizing the World Health Organization Global Breast Cancer Initiative (GBCI) framework.

**Design, Setting, and Participants:**

This cohort study used the Namibian subset of the African Breast Cancer–Disparities in Outcomes prospective cohort. Participants were all Namibian residents with confirmed incident BC who presented at the main national public oncology center of the Windhoek Central Hospital (WCH). Follow-up started from recruitment (September 8, 2014, to October 5, 2016) and ended up to 3 years after diagnosis (December 13, 2014, to September 27, 2019). Data analysis was conducted from June 2022 to August 2023.

**Exposures:**

Participants’ self-reported ethnicities were aggregated into 3 population groups: Black, mixed ancestry, and White.

**Main Outcomes and Measures:**

Three-year overall survival (OS) was examined using Cox models, and summary statistics were used to describe women’s BC journey, including GBCI pillar key performance indicators: (1) early stage (TNM I or II) diagnosis (population benchmark ≥60%), (2) prompt diagnosis, ie, 60 days or less to first health care practitioner visit (population benchmark 100%), and (3) completion of recommended multimodal treatment (MT, ie, surgery plus chemotherapy) (population benchmark ≥80%).

**Results:**

Of 405 women, there were 300 (74%) Black (mean [SD] age, 53 [15] years), 49 (12%) mixed ancestry (mean [SD] age, 53 [7] years), and 56 (14%) White (mean [SD] age, 59 [12] years) patients. Three-year OS was lowest in Black women (60% [95% CI, 54%-66%]; mixed ancestry: 80% [95% CI, 65%-89%]; White: 89% [95% CI, 77%-95%]), who had lower prevalence of early stage diagnosis (Black: 37% [95% CI, 31%-42%]; mixed ancestry and White: 75% [95% CI, 66%-83%]) and timely diagnosis (Black: 60% [95% CI, 54%-66%]; mixed ancestry and White: 77% [95% CI, 69%-85%]), while MT completion (Black: 53% [95% CI, 46%-59%]; mixed ancestry and White: 63% [95% CI, 50%-73%]) was low in all women.

**Conclusions and Relevance:**

In this cohort study of 405 Namibian residents with BC, marked racial disparities in survival were paralleled by inequities all along the BC journey. To improve BC survival, interventions are needed to promote earlier diagnosis in Black Namibian women and to increase MT initiation and completion in all women.

## Key Points

**Question:**

What are the priorities for strengthening the breast cancer (BC) journey in Namibia?

**Findings:**

In this cohort study of 405 Namibian women with incident BC recruited at the main national public oncology center, Black women were disadvantaged all along their BC journey compared with their mixed ancestry and White counterparts. There was a statistically significantly lower overall survival 3 years after BC diagnosis in Black women (60%) vs mixed ancestry and White women (85%).

**Meaning:**

These findings suggest that improvements that address the prevailing racial disparities in survival are needed across the whole BC journey to reduce BC mortality in Namibia.

## Teaser

This cohort study describes the breast cancer journey of Namibian women by race using the World Health Organization Global Breast Cancer Initiative framework.

## Introduction

Breast cancer (BC) is the leading female cancer in Namibia (approximately 30% of incident cancers and approximately 20% of cancer deaths in women in 2020).^1,2^ This Southern African upper-middle income country, whose capital is Windhoek, has a low population density, constituted of Black African individuals (approximately 87%, with approximately 50% of Ovambo ethnicity), mainly in the north, and smaller groups of mixed ancestry or White individuals (approximately 6%-7% each).^3,4,5^ Inequalities in economic development and access to services remain high.^4^ To reduce BC mortality in Namibia, identifying gaps across the entire BC journey could help prioritize effective interventions. We analyze this journey utilizing the recently launched World Health Organization Global Breast Cancer Initiative (GBCI) framework’s 3 pillars: health promotion for early detection (ie, TNM I or II diagnosis, with population benchmark of ≥60%), timely diagnosis (ie, ≤60 days to first health care practitioner [HCP] visit in the present analysis, with population benchmark of 100%), and comprehensive management (ie, completion of recommended multimodal treatment [MT, ie, surgery plus chemotherapy in this analysis], with population benchmark of ≥80%).^6^

In Namibia, there is no national BC screening program.^7^ The BC journey to diagnosis typically involves presentation to the health system with symptoms and getting biopsied after referral to a hospital where diagnostic workup can be undertaken. Histological confirmatory diagnosis is mainly obtained in the public sector provided by the Namibian Institute of Pathology, which has more than 40 laboratories across the country. Triple receptor determination is done routinely. During 2015 to 2017, therapeutic options included endocrine therapy and surgery at secondary and tertiary hospitals, chemotherapy in Windhoek and in the north, and radiotherapy in Windhoek alone. Access to trastuzumab was limited. After initial treatment, monitoring includes regular check-ups multiple times a year for the first 5 years, and once a year afterwards. The Universal Health Coverage index is among the highest in Africa, with no or little out-of-pocket costs for BC diagnosis and treatment. Public schemes exist to provide funds and free transportation and accommodation to patients with BC during treatment.^8^

Namibia is one of the countries that participated in the African Breast Cancer–Disparities in Outcomes (ABC-DO) prospective hospital-based cohort study, which collected comprehensive data on the full BC journey.^9^ Previous ABC-DO publications have focused on individual segments of the BC journey.^10,11,12,13,14^ The present article analyzes the entire journey—from BC symptom recognition to diagnosis and through treatment—of Namibian women with BC who reached the main national public oncology center. We assess the extent to which GBCI pillars key performance indicator (KPI) benchmarks have been achieved to inform where and how the BC journey can be strengthened to ultimately reduce BC mortality in Namibia.

## Methods

### Study Design and Participants

Full ABC-DO details are provided elsewhere.^9^ Between September 8, 2014, and October 5, 2016, 504 women with confirmed incident BC who presented to the AB May Cancer Centre of the Windhoek Central Hospital (WCH) were recruited into ABC-DO. This center is the largest referral public hospital and hosts the medical oncology and the only radiation oncology department in Namibia. This analysis was restricted to 405 Namibian residents (95% of women were diagnosed between June 19, 2014, and July 14, 2016) (eMethods 1 in Supplement 1). Ethical approval was obtained from all institutional ethics committees, and informed consent was obtained from all participants.^14^ This manuscript follows the Strengthening the Reporting of Observational Studies in Epidemiology (STROBE) guidelines.^15^

### Data Sources

At enrollment, women’s sociodemographic characteristics, including self-reported ethnicity, BC knowledge and beliefs, and details of their prior BC journey (dates of first symptom and dates of and travel times to previous visits to HCPs) were obtained in a nurse-led structured face-to-face interview.^12,16^ Residential addresses were geocoded, from which travel times to reach WCH were estimated via geospatial methods in AccessMod version 5.7.8 (World Health Organization).^11,17^ TNM stage at diagnosis and tumor subtype were extracted from medical and pathology records.^10^ Treatment and women’s vital status were updated from medical records and trimonthly follow-up telephone interviews with the patient or her next-of-kin.^13,18^ Among women diagnosed with nonmetastatic BC who survived at least 6 months after diagnosis, treatment indication, completion, and abandonment were defined as in the National Comprehensive Cancer Network Harmonized Guidelines.^13,19^

### Statistical Analysis

To avoid sparse data, we aggregated Namibia’s 14 regions into 5 macroregions (Central, Northern, Eastern, Southern, and Western), and ethnic groups into Black, mixed ancestry, and White (eMethods 2 and eFigure 1 in Supplement 1).^20^ Three-year overall survival (OS) was calculated on a time-since-diagnosis scale, with the date of diagnosis defined per the European Network for Cancer Registries recommendations (eMethods 2 in Supplement 1).^21^ Time-at-risk commenced on the date of (1) baseline interview or (2) diagnosis, whichever was later, and ended on the date of (1) death, (2) when the patient was last known to be alive, or (3) 3 years after diagnosis, whichever came first. We performed survival analyses by population group and macroregion of residence using Cox regression models. We present summary statistics for each indicator (including GBCI pillar KPI) (eTable 1 and eFigure 2 in Supplement 1) and used logistic regression models to identify potential determinants of long (1) precontact, (2) diagnostic, and (3) treatment intervals. Sensitivity analyses included (1) estimating 3-year OS, first by population group in women living in the Central macroregion and by macroregion in Black women only; and (2) estimating GBCI pillar 3 KPI (ie, MT completion) among women for whom MT was indicated, women with a negative or unknown HIV status, and women aged 75 years and younger to check for potential treatment contraindications not reflected in our data.^22^ All analyses were performed in Stata version 17 (StataCorp). Data analysis was conducted from June 2022 to August 2023.

## Results

### Characteristics of Patients With BC

Of 405 women, 300 (74%) were Black (43% of Ovambo ethnicity), 49 (12%) were mixed ancestry, and 56 (14%) were White (eTable 2 in Supplement 1). Black and mixed ancestry women were approximately 6 years younger at BC diagnosis than White women (mean [SD] age, Black women, 53 [15] years; women with mixed ancestry, 53 [7] years; White women, 59 [12] years). Comparing ethnic groups, Black women had the lowest educational level compared with mixed ancestry and White women (none or primary school level: 169 [56%]; 18 [37%]; 1 [2%]), most median (IQR) children at home (3 [1-4]; 2 [0-3]; 0 [0-1]), and were more likely to live in rural areas (145 [48%]; 8 [16%]; 2 [4%]), to be single (132 [44%]; 4 [7%]; 10 [20%]), or to be HIV positive (48 [16%]; 4 [8%]; 0). While most Black (130 [43%]), mixed ancestry (24 [49%]), and White (15 [48%]) women lived in Northern, Southern, and Central macroregions, respectively, this differed according to ethnicity in Black women (eTable 3 in Supplement 1).

### Survival After a BC Diagnosis

Three-years after diagnosis, 119 Black (40%), 10 mixed ancestry (20%), and 6 White (11%) women had died (Table 1). Mortality rates were 2- to 4-fold lower in mixed ancestry and White women than in Black women (mixed ancestry: HR, adjusted for age and HIV status, 0.44 [95% CI, 0.23-0.85]; White: HR, adjusted for age and HIV status, 0.23 [95% CI, 0.10-0.52]), and tended to be higher in residents of the Eastern macroregion than in those living closer to WCH (ie, Central macroregion) (HR adjusted for age and HIV status, 1.54 [95% CI, 0.86-2.75]) (Figure, A and Table 1; eFigure 3 in Supplement 1). Results remained unchanged when restricting this analysis to Central region residents or Black women (Table 1; eFigure 4 in Supplement 1).

**Table 1.** One- and 3-Year Crude OS After Breast Cancer Diagnosis, by Population Group and Macroregion in ABC-DO

Group or region	Included women, No.	Women who died before 3 y, No.	Women censored before 3 y, No.	1-y Survival	3-y Survival	3-y Mortality, HR (95% CI)^a^	Absolute survival difference at 3 y, %
**Main analysis**							
ABC-DO Namibia overall	405	135	5	88.9 (85.4-91.6)	66.5 (61.7-70.9)	NA	NA
Population group							
Black	300	119	3	86.0 (81.5-89.5)	60.2 (54.4-65.5)	1 [Reference]	0
Mixed ancestry	49	10	0	93.9 (82.2-98.0)	79.6 (65.4-88.5)	0.44 (0.23-0.85)	19.4
White	56	6	2	100	89.1 (77.3-94.9)	0.23 (0.10-0.52)	28.9
Macroregion, all women^b^							
Central	102	29	3	89.2 (81.4-93.9)	71.3 (61.4-79.1)	1 [Reference]	0
Northern	134	57	2	85.1 (77.8-90.1)	57.2 (48.4-65.1)	1.13 (0.70-1.82)	−14.1
Eastern	47	20	0	85.1 (71.3-92.6)	57.4 (42.1-70.1)	1.54 (0.86-2.75)	−13.8
Southern	71	17	0	93.0 (83.9-97.0)	76.1 (64.3-84.4)	0.75 (0.41-1.38)	4.8
Western	51	12	0	96.1 (85.2-99.0)	76.5 (62.3-85.9)	0.67 (0.34-1.31)	5.2
**Sensitivity analyses**							
Population groups, central region only							
Black	60	23	2	81.7 (69.3-89.4)	61.3 (47.7-72.3)	1 [Reference]	0
Mixed ancestry	15	3	0	80.0 (50.0-93.1)	80.0 (50.0-93.1)	0.48 (0.14-1.63)	18.8
White	27	3	1	100	88.7 (68.9-96.2)	0.30 (0.09-1.04)	27.4
Macroregion, Black women^b^							
Central	60	23	2	81.7 (69.3-89.4)	61.3 (47.7-72.3)	1 [Reference]	0
Northern	130	57	1	84.6 (77.2-89.8)	56.0 (47.1-64.1)	1.10 (0.67-1.81)	−5.2
Eastern	37	18	0	83.8 (67.4-92.4)	51.4 (34.4-65.9)	1.50 (0.80-2.79)	−9.9
Southern	38	11	0	92.1 (77.5-97.4)	71.1 (53.9-82.8)	0.69 (0.33-1.41)	9.8
Western	35	10	0	94.3 (79.0-98.5)	71.4 (53.4-83.5)	0.64 (0.30-1.34)	10.2

Abbreviations: ABC-DO, African Breast Cancer–Disparities in Outcomes; HR, hazard ratio; NA, not applicable; OS, overall survival.

^a^ HRs with 95% CIs were obtained from Cox models adjusted for population group (Black, mixed ancestry, and White women), HIV status (positive or negative or unknown), and age at baseline (<40 years, 40-49 years, 50-59 years, 60-69 years, ≥70 years).

^b^ Regions were aggregated into 5 macroregions as follows: Central, Khomas (Windhoek region); Western, Kunene and Erongo; Southern, Hardap and Karas; Eastern, Omaheke and Otjozondjupa; and Northern, Omusati, Oshana, Ohangwena, Oshikoto, Kavango West and Kavango East, and Zambezi (eFigure 1 in Supplement 1).

**Figure.**
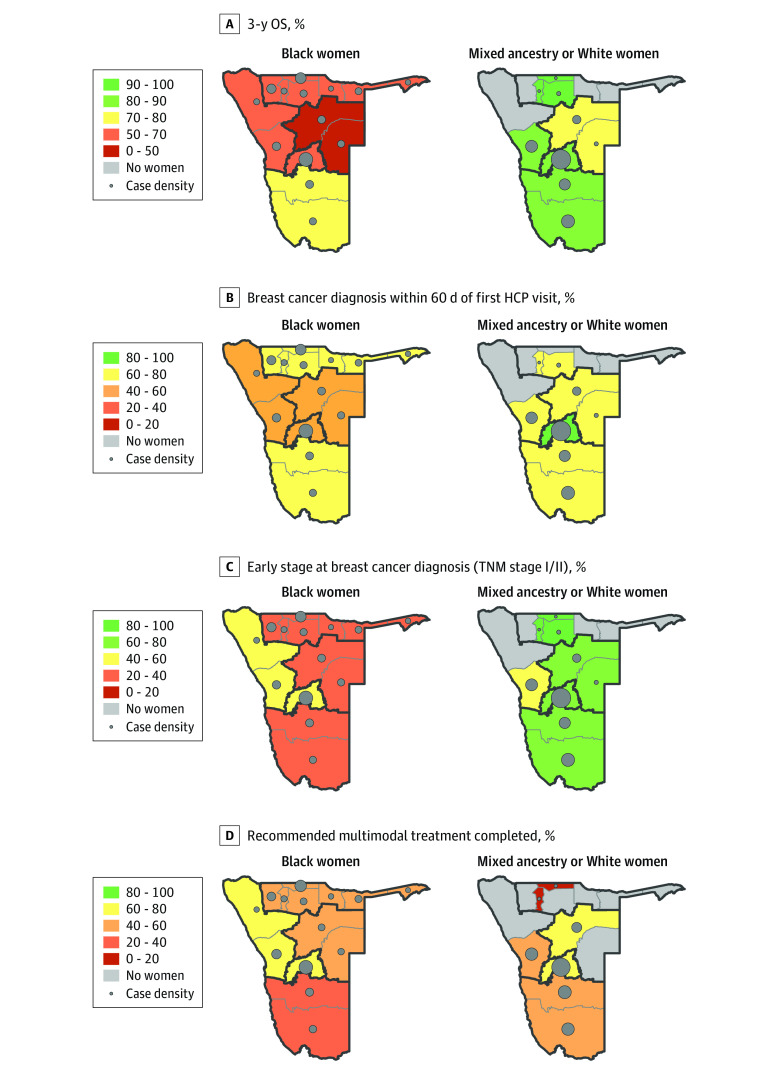
Three-Year Overall Survival (OS) After Breast Cancer Diagnosis and Global Breast Cancer Initiative (GBCI) Key Performance Indicators (KPI) in the African Breast Cancer–Disparities in Outcomes (ABC-DO) Study, by Population Group and Macroregion A, Three-year OS from breast cancer (BC) diagnosis. B, Maps displaying GBCI pillar 2 KPI, prompt BC diagnosis (benchmark ≤60 days). C, Maps displaying GBCI pillar 1 KPI (benchmark ≥60%). D, Maps displaying GBCI pillar 3 KPI, completion of recommended multimodal treatment (benchmark ≥80%). The maps in panel D represent the proportion of women with nonmetastatic breast cancer who completed surgery plus chemotherapy among those for whom these treatment modalities were indicated and who received surgery and/or chemotherapy. To avoid sparse data regions were aggregated into 5 macroregions as follows: Eastern, Omaheke and Otjozondjupa; Northern, Omusati, Oshana, Ohangwena, Oshikoto, Kavango West and Kavango East, and Zambezi; Western, Kunene and Erongo; Southern, Hardap and Karas; and Central, Khomas (where Windhoek, the country’s capital, is located) (eFigure 1 in Supplement 1). GBCI framework KPI estimates were calculated at the macroregion level. The relative contribution of each region to country total for each population group (Black and mixed ancestry and White Namibian women) is represented by case density (ie, proportion of women from a population group living in a specific region).

### Precontact Interval

The precontact interval was defined as the time from the patient first noticing symptoms to their first visit to an HCP. Most Black women had heard about BC (255 [85%]) and believed it was curable (221 [74%]) (both approximately 100% for mixed ancestry and White women), but few women, regardless of ethnicity, interpreted their symptom(s) as a possible BC (Black, 40 [13%]; mixed ancestry, 13 [27%]; White, 16 [29%]) (Table 2). Self-perceived barriers to first visit to an HCP were more common in Black women than in mixed ancestry and White women (103 [35%]; 1 [2%]; 6 [11%]). Difficulty in accessing health care was the most reported barrier (Black, 76 [26%]; mixed ancestry, 0; White, 3 [6%]). After noticing symptoms, Black women had longer median (IQR) precontact intervals than mixed ancestry and White women (41 [4-196] days; 20 [3-70] days; 10 [1-65] days; >90 days: 114 [39%]; 10 [20%]; 11 [22%]) (Table 2; eFigure 5A and eTable 4 in Supplement 1). The proportion of long precontact intervals (ie, >90 days) was higher among women with lower educational level, those who did not interpret their symptoms as a suspicious BC, or those who reported barriers to access health care, but lower in women with comorbidities, which may explain the racial disparities observed. A longer precontact interval was also associated with advanced stage at diagnosis (eTable 4 in Supplement 1).

**Table 2.** Characteristics of the Precontact Interval From First Noticed Symptom(s) to First Visit to an HCP in Namibia in African BC–Disparities in Outcomes Study, by Population Group

Factor	Women, No. (%)		
	Black (n = 300)	Mixed (n = 49)	White (n = 56)
BC awareness			
Heard about BC	255 (85.0)	49 (100)	56 (100)
Believed BC is potentially curable	221 (73.7)	48 (98.0)	55 (98.2)
Interpretation of first symptom(s) as a possible BC	40 (13.3)	13 (26.5)	16 (28.6)
Barriers to first visit to an HCP			
Self-reported travel time from home to first visit to an HCP, median (IQR), min	26 (15-60)	15 (10-25)	10 (9-17)
Transportation from home to first visit to an HCP			
Private car	96 (32.0)	23 (46.9)	52 (92.9)
Public transportation	100 (33.3)	6 (12.2)	1 (1.8)
Walking	100 (33.3)	19 (38.8)	2 (3.6)
Other or unknown	4 (1.3)	1 (2.0)	1 (1.8)
Barriers to access first visit to an HCP^a^			
Access to care barrier^b^	76 (25.7)	0	3 (5.6)
Belief barrier^c^	17 (5.7)	1 (2.0)	3 (5.6)
Lack of time	11 (3.7)	0	1 (1.9)
Husband	1 (0.3)	0	0
Other	11 (3.7)	0	3 (5.4)
At least 1 barrier	103 (34.8)	1 (2.0)	6 (11.1)
Precontact interval, median (IQR), d^d^	41 (4-196)	20 (3-70)	10 (1-65)

Abbreviations: BC, breast cancer; HCP, health care practitioner (formal or informal).

^a^ Six women with errors in first visit information were excluded from this analysis.

^b^ Difficulties obtaining an appointment, access transportation, or a lack of ability to pay for the treatment.

^c^ Belief barrier encompassed embarrassment, fear of rejection by the husband or family members, fear of being unwell or dying, belief that treatment is pointless, lack of trust in medical doctors or other health professionals, and preference for traditional medicine.

^d^ Twelve women with errors in the date of their first visit (n = 6) or in the date of first noticing symptoms (n = 9) were excluded from this analysis.

### Diagnostic Interval

The diagnostic interval was defined as the time between first visit to an HCP and BC diagnosis. A large majority of mixed ancestry and White women first consulted a primary-level HCP (41 [84%]; 47 [87%]) to seek help, whereas Black women first visited either a first-level (171 [58%]) or secondary or tertiary-level hospital (122 [41%]) (Table 3). BC was suspected for approximately 25% of women at the first visit, similar across population groups, but inappropriate referrals were twice as likely in Black (87 [29%]) and mixed ancestry (12 [25%]) women than in White women (7 [13%]). On average, 2 visits were required before referral for diagnosis, across ethnicity (Table 3). The median (IQR) diagnostic interval was substantially longer in Black and mixed ancestry than in White women (31 [0-136] days; 23 [4-90] days; 8 [0-22] days; GBCI pillar 2 KPI benchmark of ≤60 days reached for 178 Black women [60%]; 35 mixed ancestry women [71%]; 45 White women [83%]) (Table 3 and Figure, B). All participants underwent a tumor biopsy and nearly all had a known tumor subtype (Black, 291 [97%]; mixed ancestry, 46 [94%]; White, 56 [100%]). After the first visit, time to biopsy was longer for Black women (≤1 month: Black, 120 [41%]; mixed ancestry and White, 63 [61%]; >6 months: Black, 75 [26%]; mixed ancestry and White, 16 [16%]) (eFigure 5B in Supplement 1). Delayed diagnosis (ie, >60 days) was associated with being Black, having shorter precontact interval (ie, ≤90 days), and reporting barriers to health care access and was inversely associated with living with a partner (eTable 5 in Supplement 1). Nationally, the percentage of Black women diagnosed at early stages was half that in other groups (TNM stages I or II: Black, 110 [37%]; mixed ancestry, 37 [76%]; White, 42 [75%]) (Table 3). While the GBCI pillar 1 KPI benchmark of 60% early-stage diagnosis was met for mixed ancestry and White women from most macroregions, it was not reached for Black women from any macroregion (Figure, C).

**Table 3.** Characteristics of the Diagnostic Interval From First Visit to an HCP to BC Diagnosis in Namibia in African BC–Disparities in Outcomes Study, by Population Group

Variable	Women, No. (%)		
	Black (n = 300)	Mixed (n = 49)	White (n = 56)
**Diagnostic pathway**			
Screen-detected	4 (1.3)	5 (10.2)	12 (21.4)
Type of first HCP visited			
Primary care	171 (57.8)	41 (83.7)	47 (87.0)
Secondary or tertiary	122 (41.2)	8 (16.3)	6 (11.1)
Informal	3 (1.0)	0	1 (1.9)
Outcome of first contact			
Inappropriate outcome	87 (29.4)	12 (24.5)	7 (13.0)
BC suspected	78 (26.4)	12 (24.5)	15 (27.8)
Patient referred to another practitioner or facility	130 (43.9)	25 (51.0)	32 (59.3)
Not applicable or unknown	1 (0.3)	0	0
No. of HCP visits before referral for diagnosis, median (IQR)	2 (1-3)	2 (1-3)	2 (1-2)
Biopsy undergone	313 (100)	56 (100)	36 (100)
Diagnostic interval^a^^,^^b^			
Median (IQR), in days	31 (0-136)	23 (4-90)^c^	8 (0-22)^d^
≤60 d	178 (60.1)	35 (71.4)^c^	45 (83.3)^d^
Time interval from biopsy to pathology report, median (IQR), d^e^	8 (5-14)	6 (3-11)	7 (4-11)
**BC characteristics at diagnosis**			
Known TNM stage	300 (100)	49 (100)	56 (100)
TNM stage at diagnosis			
Early stage at diagnosis^b^	110 (36.7)	37 (75.5)^d^	42 (75.0)^d^
TNM stage III	143 (47.7)	9 (18.4)	10 (17.9)
TNM stage IV	47 (15.7)	3 (6.1)	4 (7.1)
Known tumor subtype	291 (97.0)	46 (93.9)	56 (100)

Abbreviations: BC, breast cancer; HCP, health care practitioner (formal or informal).

^a^ Six women with incorrect dates of first visit to an HCP were excluded from this analysis.

^b^ Global Breast Cancer Initiative pillars 1 and 2 key performance indicators were compared between racial groups (mixed ancestry vs Black women and White vs Black women). *P* values were obtained from Wilcoxon test (diagnostic interval as continuous) or χ^2^ test (diagnostic interval ≤60 days vs >60 days; early vs late stage at diagnosis).

^c^ *P* ≥ .05.

^d^ *P* < .001.

^e^ Two women with unknown date of pathology report were excluded from this analysis.

### Treatment Interval and BC Management of Nonmetastatic Disease

Median treatment interval was approximately 1 month, similar among population groups (Table 4). After biopsy, median time to pathology report was approximately 1 week, with approximately 95% of patients receiving the report in less than 1 month (Table 3; eFigure 5C in Supplement 1). Black women lived further away from WCH (≥500 km: Black, 124 [41%]; mixed ancestry, 6 [12%]; White, 3 [5%]) and relied more on transportation provided by the Cancer Association of Namibia or the hospital (Black, 179 [60%]; mixed, 13 [27%]; White, 3 [5%]) to reach WCH (eFigure 6 in Supplement 1). Hence, after receiving their report, time to reach WCH was longer in Black women (>1 month: Black, 33 [24%]; mixed ancestry and White, 5 [9%]); however, once there, treatment was initiated shortly (≤2 weeks: Black, 135 [93%]; mixed ancestry and White, 45 [76%]) (eFigure 5D and 5E in Supplement 1). Delayed treatment initiation was associated with lower educational level and difficulties accessing health care, which may explain the racial differences observed (eTable 6 in Supplement 1).

**Table 4.** Characteristics of the Treatment Interval From BC Diagnosis to Treatment Initiation and BC Management in Namibia in African BC–Disparities in Outcomes^a^

Treatment	Women, No. (%)		
	Black (n = 247)	Mixed (n = 46)	White (n = 52)
**Among women with a known treatment status**			
No.	240	45	52
Any treatment received	235 (97.9)	45 (100)	52 (100)
Treatment indicated			
Surgery	240 (100)	45 (100)	52 (100)
Chemotherapy	236 (98.3)	39 (86.7)	38 (73.1)
Endocrine therapy	180 (75.0)	31 (68.9)	46 (88.5)
Radiotherapy	177 (73.8)	22 (48.9)	27 (51.9)
**Among women for whom treatment is indicated per NCCN Harmonized guidelines**			
Treatment received, No./total No. (%)			
Surgery	177/240 (73.8)	37/45 (82.2)	49/52 (94.2)
Chemotherapy	186/236 (78.8)	34/39 (87.2)	30/38 (78.9)
Endocrine therapy	174/180 (96.7)	30/31 (96.8)	44/46 (95.7)
Radiotherapy	114/177 (64.4)	16/22 (72.7)	19/27 (70.4)
MT (ie, surgery plus chemotherapy)	149/236 (63.1)	29/39 (74.4)	28/38 (73.7)
Treatment interval			
Median (IQR), d	33 (21-58)	30 (11-42)^b^	31 (14-66)^b^
≤30 d	105/235 (44.7)	24/45 (53.3)^b^	26/52 (50.0)^b^
**Among women who received their recommended surgery and/or chemotherapy**			
No.	212	36	37
MT completed^c^			
Yes			
MT initiated and chemotherapy completed^d^	112 (52.8)	22 (61.1)^b^	23 (62.2)^b^
Among completed, timely initiated^e^	57 (26.9)	15 (41.7)	13 (35.1)
Among completed, initiation delayed^e^	55 (25.9)	7 (19.4)	10 (27.0)
No			
MT initiated but chemotherapy not completed^c^	37 (17.5)	7 (19.4)^b^	5 (13.5)^b^
Chemotherapy ended before completion	33 (15.6)	6 (16.7)	4 (10.8)
Woman died within 6 mo of chemotherapy initiation	0	0	0
Chemotherapy completion unknown	4 (1.9)	1 (2.8)	1 (2.7)
MT not initiated^d^	63 (29.7)	7 (19.4)^b^	9 (24.3)^b^
Chemotherapy not initiated	26 (12.3)	2 (5.6)	7 (18.9)
Surgery not received	37 (17.5)	5 (13.9)	2 (5.4)

Abbreviations: BC, breast cancer; MT, multimodal treatment; NCCN, National Comprehensive Cancer Network.

^a^ All analyses were performed in women with nonmetastatic BC who were still alive 6 months after BC diagnosis.

^b^ *P* ≥ .05.

^c^ Greater than 85% of the total cumulative chemotherapy completed defined as 5 or more cycles of fluorouracil, doxorubicin, and cyclophosphamide therapy (or equivalent) administered within 15 weeks of chemotherapy initiation or 7 or more cycles of fluorouracil, doxorubicin, cyclophosphamide, and taxane therapy within 28 weeks, after the first dose or cycle (the recommended time frame is within 24 weeks of diagnosis).

^d^ Global Breast Cancer Initiative pillar 3 key performance indicator was compared between racial groups (mixed ancestry vs Black women and White vs Black women). *P* values were obtained from Wilcoxon test (treatment interval as continuous) or χ^2^ test (treatment interval ≤30 days vs >30 days; MT initiated and chemotherapy completed, yes vs no; MT initiated but chemotherapy not completed, yes vs no; MT not initiated, yes vs no).

^e^ Treatment considered timely initiated if surgery was performed or chemotherapy was initiated within 30 days of BC diagnosis.

MT indication was high (Black, 236 of 240 with known treatment status [98%]; mixed ancestry, 39 of 45 with known treatment status [87%]; White: 38 of 52 with known treatment status [73%]), but Black women were less likely to receive it (Black, 149 of 236 [63%]; mixed ancestry, 29 of 39 [74%]; White, 28 of 38 [74%]). Once initiated, the proportion of women who completed their initiated MT was low (Black, 112 of 212 [53%]; mixed ancestry, 22 of 36 [61%]; White, 23 of 37 [62%]), and very few completed it in a timely manner (ie, surgery or chemotherapy within 30 days of diagnosis; Black, 57 [27%]; mixed ancestry, 15 [42%]; White, 13 [35%]) (Table 4 and Figure). None of the macroregions reached the GBCI pillar 3 KPI benchmark of 80% or greater. MT abandonment was similar among population groups (Black, 33 [16%]; mixed ancestry, 6 [17%]; White, 4 [11%]) (Table 4). Results remained similar when restricting this analysis to women 75 years or younger; with negative or unknown HIV status; or for whom MT was indicated, irrespective of initiation (eTable 7 in Supplement 1).

## Discussion

### Main Findings

We observed marked racial disparities in survival after a BC diagnosis in this cohort of Namibian women attending a tertiary oncological center in Windhoek, paralleled by racial inequities in accessing BC care. GBCI pillars 1 and 2 KPI were worse for Black than for mixed ancestry and White women, irrespective of their macroregion of residence, while GBCI pillar 3 KPI was low for all population groups. Despite faring better, mixed ancestry and White women only achieved 1 of the 3 KPI benchmarks (ie, early-stage diagnosis). To avert BC deaths in Namibia, improvements are needed in all GBCI framework pillars.

### Interpretation

Three-year OS estimates were suboptimal for all population groups, far below the 5-year survival observed in high-income countries (approximately 90%).^23^ Another study by the African Cancer Registry Network based on 64 Namibian women estimated a higher 3-year survival than ABC-DO, at 79%.^24^ However, there was no racial stratification, 15% of the selected sample had no follow-up data, and 13% of women were lost to follow-up. In ABC-DO, within the Central macroregion where WCH is located, racial disparities in survival were similar to the all-region analyses, suggesting persisting racial inequities in accessing BC diagnosis and treatment, consistent with the later stage at diagnosis and relatively lower MT completion rate observed in Black women.

The higher proportion of late stage at diagnosis in Black women was likely driven by longer precontact and diagnostic intervals.^10,12^ Patients’ low BC awareness may have been a major determinant of longer precontact interval. However, once a patient had presented, delayed diagnosis resulted from missed opportunities for referrals, as observed in women with shorter precontact intervals who likely exhibited more subtle symptoms, suggesting low BC awareness by HCPs. Hence, some patients with BC with the greatest potential for receiving potentially curative treatment may be subsequently diagnosed too late. Many women with a long precontact interval experienced difficulties in accessing an HCP (eg, in getting an appointment or lack of transportation). Interestingly, women with comorbidities—likely used to frequent contacts with HCPs—had shorter precontact intervals, suggesting that some women may not know where to go when they notice symptoms. However, they did not have shorter diagnostic interval or earlier stage at diagnosis, suggesting that after first presentation, they experienced the same difficulties in navigating the system as other women.^25^ These factors accounted for the racial disparities in precontact interval. However, adjusting for the identified determinants of long diagnostic interval (ie, social support, precontact delays, and health care navigation barriers) did not fully explain the higher risk of diagnostic delays observed in Black or mixed ancestry women. This could be partly explained by inequitable access to health care and variability in its quality (ie, available infrastructure, staff, training) depending on the macroregion of residence (ie, Black women mostly live in the North and remotely), unmeasured confounding, or possible true persisting racial inequities in accessing care in Namibia.

In addition to stage at diagnosis, quality of BC care is also a major driver of BC survival.^13,14^ All patients with nonmetastatic BC should at least receive MT (ie, surgery plus chemotherapy). Furthermore, prompt initiation and completion of MT are both essential to not compromise survival.^14,22^ The GBCI pillar 3 KPI benchmark of at least 80% MT completion accounts for barriers to access and afford standard BC treatment, which are common in low- and middle-income countries.^22^ In our study, MT completion was far lower than 80% for all population groups despite the existing support schemes to remove such barriers in Namibia and the absence of drug shortages at WCH. Indirect high financial toxicity through a woman’s job loss or the need to care for children remains possible, contributing to the overall low treatment completion observed. In Black women, most treatment delays were likely caused by longer times to reach WCH, thus highlighting potential difficulties for some patients to navigate the system or to overcome barriers to access WCH. Consequently, missed opportunities to treat a potentially initially curable BC may arise. In mixed ancestry and White women, most treatment delays occurred after presentation to WCH, possibly because of medical decisions. Interestingly, once MT was initiated, there was no difference in chemotherapy abandonment across population groups (approximately 15%). However, our analysis focused on the first chemotherapy course only, and reasons for not initiating or abandoning treatment were not documented. Hence, we could not differentiate between abandonment by the woman (including refusals), issues in accessing health care, or medical decisions, which may have resulted in underestimating treatment completion.^13,18^

### Future Implications

Availing of the excellent diagnostic services and good access to MT in Namibia, shortening of the precontact diagnostic intervals, and strengthening MT completion rates appear as the main priorities to reduce BC mortality in Namibia. Context-specific interventions to promote early diagnosis in Black women are also needed. This could be achieved by raising BC awareness in the community and among HCPs to ensure early recognition of symptoms and timely referral for diagnosis. Comprehensive women’s health clinics with an educative role could be set up to target both elimination of cervical cancer and early detection of BC. Improving patient navigation is also required by, for instance, providing clear guidance to HCPs and the women regarding where they should be referred for diagnosis and treatment and information on existing schemes to overcome barriers.^26^ This information should be available in multiple languages and made accessible to all, including illiterate women. New technologies (eg, mHealth) could play a role in helping women through treatment to improve completion.^27,28^ ABC-DO was pre–COVID-19 pandemic, and since its implementation, an oncology center in the North and a breast clinic in Windhoek have been set up. Hence, future studies are needed to evaluate the impact of these recent developments and that of COVID-19 on BC outcomes in Namibia.

### Limitations

This study has limitations. As recruitment was hospital-based and monocentric, our cohort is not representative of all patients with BC in Namibia; the private sector and women not referred to or unable to access WCH were not included (ie, mostly Black women living further away from Windhoek). Hence, examining geographical disparities in BC care was not possible, and at the population level, disparities in survival are likely underestimated and expected to increase with distance from Windhoek. As WCH is a chemotherapy center, women would be referred there for chemotherapy, hence the very high rate of chemotherapy indication in our sample. We reported OS instead of BC-specific survival as cause of death was difficult to ascertain; however, BC mortality is high in low- and middle-income countries, and most women who died would have died from this disease.

## Conclusions

We identified marked racial disparities in survival after a BC diagnosis in Namibia, which were underpinned by inequities in access to cancer care as highlighted by the suboptimal GBCI framework pillars KPI estimates. Our results give clear guidance on which interventions are needed to promote early diagnosis and improve MT access and completion to reduce BC mortality in Namibia.

## Notes

Supplement 1. **eMethods 1.** Study Design and Participants **eMethods 2.** Statistical Analysis **eFigure 1.** Map of Namibia Showing Region Labels and Macroregions Used in the Analysis **eTable 1.** Indicators Used in Present ABC-DO Analysis **eFigure 2.** BC Journey in Namibia in the ABC-DO Study **eTable 2.** Characteristics of Included BC Patients From ABC-DO Namibia **eTable 3.** Distribution of Ethnic Groups by Macroregion of Residence in ABC-DO in Namibia **eFigure 3.** Crude Kaplan-Meier Curves of OS After a BC Diagnosis in ABC-DO Women by Population Group and Macroregion **eFigure 4.** Crude Kaplan-Meier OS Curves After BC Diagnosis in Black Women, by Ethnic Group and Macroregion **eFigure 5.** Breast Cancer Journey in Women Included in ABC-DO in Namibia **eTable 4.** Characteristics of Women With Long vs Shorter Precontact Interval in Namibia in ABC-DO **eTable 5.** Characteristics of Women With Long vs Shorter Diagnostic Interval in Namibia in ABC-DO **eFigure 6.** Geographical Barriers to Access Health Care in Namibia in ABC-DO **eTable 6.** Characteristics of Women With Long vs Shorter Treatment Interval From Diagnosis in Namibia in ABC-DO **eTable 7.** Sensitivity Analysis Regarding Recommended Multimodal Treatment (Surgery Plus Chemotherapy) Completion in Namibia in ABC-DO
eReferences.

Supplement 2.
Data Sharing Statement
