# Supplementary material for: Analysis of the Breast Cancer Journey in Namibia
Source: JAMA Netw Open. 2023 Nov 3;6(11):e2341402. doi: 10.1001/jamanetworkopen.2023.41402 (PMC10625043; doi:10.1001/jamanetworkopen.2023.41402)
Supplement: Supplement 1. — eMethods 1. Study Design and Participants eMethods 2. Statistical Analysis eFigure 1. Map of Namibia Showing Region Labels and Macroregions Used in the Analysis eTable 1. Indicators Used in Present ABC-DO Analysis eFigure 2. BC Journey in Namibia in the ABC-DO Study eTable 2. Characteristics of Included BC Patients From ABC-DO Namibia eTable 3. Distribution of Ethnic Groups by Macroregion of Residence in ABC-DO in Namibia eFigure 3. Crude Kaplan-Meier Curves of OS After a BC Diagnosis in ABC-DO Women by Population Group and Macroregion eFigure 4. Crude Kaplan-Meier OS Curves After BC Diagnosis in Black Women, by Ethnic Group and Macroregion eFigure 5. Breast Cancer Journey in Women Included in ABC-DO in Namibia eTable 4. Characteristics of Women With Long vs Shorter Precontact Interval in Namibia in ABC-DO eTable 5. Characteristics of Women With Long vs Shorter Diagnostic Interval in Namibia in ABC-DO eFigure 6. Geographical Barriers to Access Health Care in Namibia in ABC-DO eTable 6. Characteristics of Women With Long vs Shorter Treatment Interval From Diagnosis in Namibia in ABC-DO eTable 7. Sensitivity Analysis Regarding Recommended Multimodal Treatment (Surgery Plus Chemotherapy) Completion in Namibia in ABC-DO eReferences. [file jamanetwopen-e2341402-s001.pdf]

Supplemental Online Content

Boucheron P, Zietsman A, Pontac J, et al. Analysis of the breast cancer journey in Namibia. *JAMA Netw Open.* 2023;6(11):e2341402. doi:10.1001/jamanetworkopen.2023.41402

eMethods 1. Study Design and Participants

eMethods 2. Statistical Analysis

eFigure 1. Map of Namibia Showing Region Labels and Macroregions Used in the Analysis

eTable 1. Indicators Used in Present ABC-DO Analysis

eFigure 2. BC Journey in Namibia in the ABC-DO Study

eTable 2. Characteristics of Included BC Patients From ABC-DO Namibia

eTable 3. Distribution of Ethnic Groups by Macroregion of Residence in ABC-DO in Namibia

eFigure 3. Crude Kaplan-Meier Curves of OS After a BC Diagnosis in ABC-DO Women by Population Group and Macroregion

eFigure 4. Crude Kaplan-Meier OS Curves After BC Diagnosis in Black Women, by Ethnic Group and Macroregion

eFigure 5. Breast Cancer Journey in Women Included in ABC-DO in Namibia

eTable 4. Characteristics of Women With Long vs Shorter Precontact Interval in Namibia in ABC-DO

eTable 5. Characteristics of Women With Long vs Shorter Diagnostic Interval in Namibia in ABC-DO

eFigure 6. Geographical Barriers to Access Health Care in Namibia in ABC-DO

eTable 6. Characteristics of Women With Long vs Shorter Treatment Interval From Diagnosis in Namibia in ABC-DO

eTable 7. Sensitivity Analysis Regarding Recommended Multimodal Treatment (Surgery Plus Chemotherapy) Completion in Namibia in ABC-DO

eReferences.

This supplemental material has been provided by the authors to give readers additional information about their work.

© 2023 Boucheron P et al. *JAMA Network Open.*

eMethods 1. Study Design and Participants

This analysis was restricted to Namibian residents (n=458) because the pre-diagnostic pathway and outcomes of medical tourists would have been influenced by their journey elsewhere. Women with prevalent BC (i.e. first diagnosis >2 years before enrolment (n=25) or who first noticed symptoms >5 years before enrolment (n=28)) were excluded (n=53), leaving 405 women in the analysis.

eMethods 2. Statistical Analysis

Individual ethnic groups (as per woman's self-report) were aggregated into three population groups by ancestry, as follows: (i) African ancestry (i.e., Caprivian, Damara, Herero, Kavango, Nama, Ovambo, San, Tswana, Xhosa) labelled "Black", (ii) European ancestry (i.e., Whites) labelled "White" and (iii) Mixed ancestry (i.e., both African and European/Asian ancestries, including Coloured and Basters) labelled "Mixed ancestry". Two women with unknown ethnic group were considered Black. The date of diagnosis was defined based on the European Network for Cancer Registries recommendations as follows, i.e., the earliest non-missing date of i) biopsy (79%), laboratory receipt (14%), pathology report (1%), and previous treatment (6%).[1]

© 2023 Boucheron P et al. *JAMA Network Open.*

eFigure 1. Map of Namibia Showing Region Labels and Macroregions Used in the Analysis

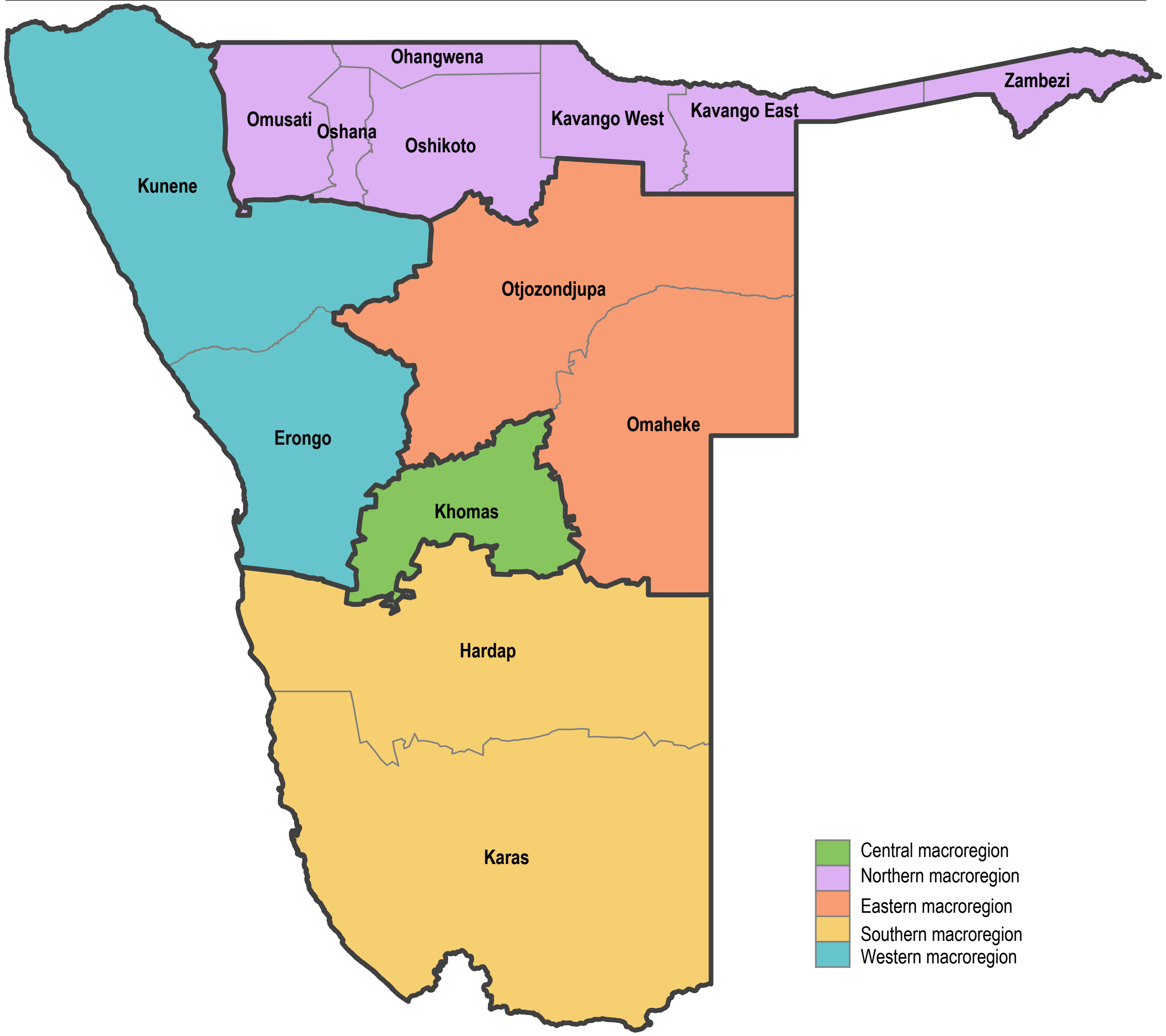

© 2023 Boucheron P et al. *JAMA Network Open.*

eTable 1. Indicators Used in Present ABC-DO Analysis

Domain	Metric
**Overarching indicators**	
Survival[a]	Population group- and geographical macroregion-specific survival 1, and 3 years after a BC diagnosis
**Pre-contact period: from first noticed symptom(s) to first visit to a HCP[a]**	
BC awareness	% BC patients who had heard about BC, believed BC is potentially curable, and interpreted their first noticed symptom(s) as a possible BC
Barriers to first visit to a HCP	% BC patients who encountered barriers to a first visit to a HCP; Median (IQR) self-reported travel time from home to first visit to a HCP, in minutes, and mean of transportation to first HCP visit
Pre-contact interval	Median (IQR) time to first visit to a HCP (i.e., date of first HCP visit - date of first noticing symptom(s)), in days. Long pre-contact interval (i.e., >90 days)
**Diagnostic period (from first visit to a HCP to BC diagnosis)[a]**	
Screen-detected	% BC patients who were screen-detected
Health education/system	Type of first HCP visited, % BC patients for whom BC was suspected by the first HCP visited, and median (IQR) no. HCP visits before BC was suspected
Diagnostic interval (GBCI pillar II KPI)	Median (IQR) time from first visit to a HCP to diagnosis, in days, and % diagnosed within 60 days of first visit. Long diagnostic interval (i.e., >60 days) (woman-level benchmark ≤60 days)
Alternative diagnostic interval measure	Median (IQR) time between biopsy and pathology report, in days, among those who underwent a biopsy (% who received a biopsy reported)
Access to immunohistochemistry	% BC patients with a known tumour subtype
Early stage at diagnosis (GBCI pillar I KPI)	% BC patients diagnosed at TNM stages I/II, among those with known stage (% with known stage reported) (population-level benchmark ≥60%)
**Treatment interval and BC management[b]**	
Known treatment status	% BC patients with a known treatment status

© 2023 Boucheron P et al. *JAMA Network Open.*

Domain	Metric
Treatment interval (GBCI pillar III KPI)[c]	Median (IQR) time from **BC diagnosis** to treatment initiation, in days, and % treated within 30 days of BC diagnosis. Long treatment interval (i.e., >30 days).
Treatment indication	% BC patients for whom each of surgery, chemotherapy, ET, radiotherapy, and MT (i.e., surgery plus chemotherapy) were indicated
Treatment received	% BC patients who received any treatment and, among indicated, % BC patients who received each of surgery, chemotherapy, ET, radiotherapy, or MT
Treatment completion (GBCI pillar III KPI)[d]	% BC patients who received timely[e] and adequately completed[d] MT, when indicated and surgery and/or chemotherapy received (population-level benchmark ≥80%)

BC: breast cancer; ET: endocrine therapy; HCP: healthcare practitioner (formal and informal); IQR: inter-quartile range; KPI: Key Performance Indicator; MT: Multimodal treatment

[a]Among all women recruited in ABC-DO in Namibia

[b]Among non-metastatic ABC-DO women recruited in Namibia who were still alive six months after BC diagnosis

[c]Among treated (any treatment)

[d]>85% of the total cumulative chemotherapy completed defined as ≥5 cycles of fluorouracil, doxorubicin, and cyclophosphamide therapy (or an equivalent regimen) administered within 15 weeks of chemotherapy initiation, or ≥7 cycles of fluorouracil, doxorubicin, cyclophosphamide, and taxane therapy within 28 weeks, after the first dose or cycle (the recommended timeframe is within 24 weeks of diagnosis)

[e]Treatment was considered timely if it was initiated within 30 days of BC diagnosis

© 2023 Boucheron P et al. *JAMA Network Open.*

eFigure 2. BC Journey in Namibia in the ABC-DO Study

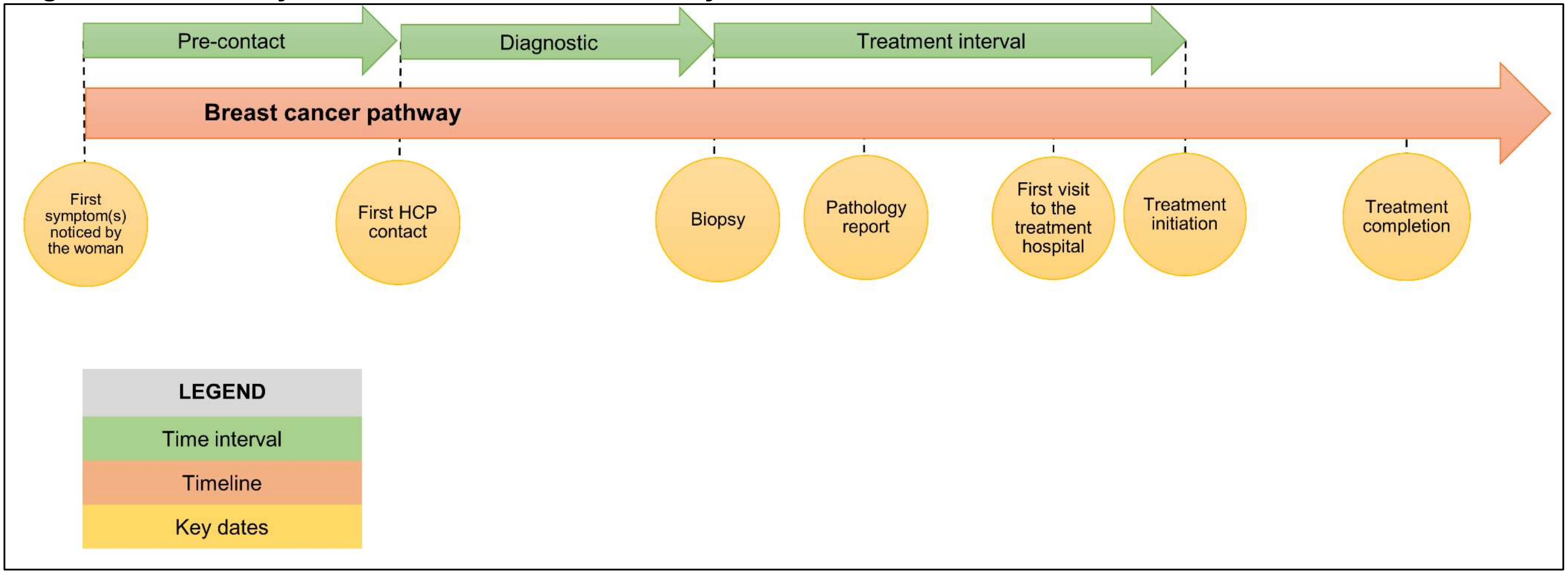

NB: BC=Breast cancer; HCP=Healthcare practitioner

© 2023 Boucheron P et al. *JAMA Network Open.*

eTable 2. Characteristics of Included BC Patients From ABC-DO Namibia

		Black (n=300)	Mixed Ancestry (n=49)	White (n=56)
		No. (%)	No. (%)	No. (%)
**Demographics**				
**Age at diagnosis**	<40	63 (21.0)	8 (16.3)	2 (3.6)
	40-49	81 (27.0)	11 (22.4)	13 (23.2)
	50-59	70 (23.3)	16 (32.7)	14 (25.0)
	60-69	48 (16.0)	8 (16.3)	15 (26.8)
	70+	38 (12.7)	6 (12.2)	12 (21.4)
	Mean age (SD)	52.5 (14.8)	53.2 (7.0)	59.1 (12.3)
**Ethnicity**	Ovambo	129 (43.0)	-	-
	Herero	49 (16.3)	-	-
	Damara	45 (15.0)	-	-
	Nama	35 (11.7)	-	-
	Kavango	22 (7.3)	-	-
	Caprivian	10 (3.3)	-	-
	San/Bushman	5 (1.7)	-	-
	Other/Unknown[a]	5 (1.6)	-	-
	Baster	-	13 (26.5)	-
	Coloured	-	36 (71.4)	-
**Macroregion**	Central	60 (20.0)	15 (30.6)	15 (48.2)
	Northern	130 (43.3)	1 (2.0)	1 (5.4)
	Eastern	37 (12.3)	3 (6.1)	3 (12.5)
	Southern	38 (12.7)	24 (49.0)	24 (16.1)
	Western	35 (11.7)	6 (12.2)	6 (17.9)
**Religion**	Christian	290 (96.7)	47 (95.9)	54 (96.4)
	Other[b]	10 (3.3)	2 (4.0)	2 (3.6)
**Education**	None/Primary school	169 (56.3)	18 (36.7)	1 (1.8)
	Secondary/high school	99 (33.0)	19 (38.8)	25 (44.6)
	Technical/University	32 (10.7)	12 (24.5)	30 (53.6)
**Socioeconomic status**	Low	151 (50.3)	0 (0.0)	0 (0.0)
	Medium	99 (33.0)	25 (51.0)	18 (32.1)
	High	50 (16.7)	24 (49.0)	38 (67.9)
	Median score (IQR) (from 1 to 9)	4 (2, 7)	7 (7, 8)	8 (7, 9)
**Residential area**	Urban	155 (51.7)	41 (83.7)	54 (96.4)

© 2023 Boucheron P et al. *JAMA Network Open.*

		Black (n=300)	Mixed Ancestry (n=49)	White (n=56)
		No. (%)	No. (%)	No. (%)
**Demographics**				
	Rural	145 (48.3)	8 (16.3)	2 (3.6)
**Marital status**	Married	99 (33.0)	34 (60.7)	26 (53.1)
	Divorced	69 (23.0)	18 (32.1)	13 (26.5)
	Single	132 (44.0)	4 (7.1)	10 (20.4)
**Live alone**	Yes	23 (7.7)	10 (17.9)	4 (8.2)
	No	277 (92.3)	46 (82.1)	45 (91.8)
**No. children at home**	Median No. (IQR)	3 (1, 4)	1 (0, 3)	0 (0, 1)
**Comorbidities**				
**HIV status**	Negative/Unknown status	252 (84.0)	45 (91.8)	56 (100)
	Positive	48 (16.0)	4 (8.2)	0 (0.0)
**BMI**	<25	129 (43.0)	14 (28.6)	20 (35.7)
	[25-30[	78 (26.0)	12 (24.5)	11 (19.6)
	30+	77 (25.7)	21 (42.9)	23 (41.1)
	Unknown	16 (5.3)	2 (4.1)	2 (3.6)
	Mean BMI (SD)	26.4 (6.8)	29.6 (7.2)	28.6 (6.5)
**Comorbidities**[c]	Yes	147 (49.0)	32 (65.3)	34 (60.7)
	No	153 (51.0)	17 (34.7)	22 (39.3)
**Tobacco**	Never user	241 (80.3)	28 (57.1)	33 (58.9)
	Ever user	59 (19.7)	21 (42.9)	23 (41.1)
**Alcohol**	Never drank	147 (49.0)	20 (40.8)	20 (35.7)
	Ever drank	153 (51.0)	29 (59.2)	36 (64.3)

BC: Breast cancer; BMI: Body Mass Index; IQR: Inter-quartile range; SD: Standard deviation
[a]Other ethnic groups included: Tswana (n=1), Xhosa (n=1), Angolan (n=1), and unknown (n=2)
[b]Other religions included Jehovah (n=5), traditional (n=3), Jewish (n=2), and none (n=4)
[c]Having ever suffered from one of the following chronic conditions: hypertension, heart disease, diabetes, chronic anaemia, chronic obstructive pulmonary disease (COPD, e.g. chronic bronchitis, emphysema), asthma, hepatitis B or C, tuberculosis, other chronic infection, other cancer, other chronic disease

© 2023 Boucheron P et al. *JAMA Network Open.*

eTable 3. Distribution of Ethnic Groups by Macroregion of Residence in ABC-DO in Namibia

		Macroregion				
		Central (Windhoek region) (n=90)	Northern (n=132)	Eastern (n=43)	Southern (n=86)	Western (n=47)
		No. (%)	No. (%)	No. (%)	No. (%)	No. (%)
**Black**	Ovambo	17 (13.2)	93 (72.1)	5 (3.9)	3 (2.3)	11 (8.5)
	Herero	16 (32.7)	4 (8.2)	18 (36.7)	2 (4.1)	9 (18.4)
	Damara	14 (31.1)	1 (2.2)	4 (8.9)	11 (24.4)	15 (33.3)
	Nama	10 (28.6)	0 (0.0)	5 (14.3)	20 (57.1)	0 (0.0)
	Kavango	0 (0.0)	20 (90.9)	2 (9.1)	0 (0.0)	0 (0.0)
	Caprivian	0 (0.0)	10 (100)	0 (0.0)	0 (0.0)	0 (0.0)
	San/Bushman	0 (0.0)	1 (20.0)	3 (60.0)	1 (20.0)	0 (0.0)
	Other ethnicities[a]	3 (60.0)	1 (20.0)	0 (0.0)	1 (20.0)	0 (0.0)
**Mixed ancestry**	Baster	5 (38.5)	0 (0.0)	0 (0.0)	7 (53.8)	1 (7.7)
	Coloured	10 (27.8)	1 (2.8)	3 (8.3)	17 (47.2)	5 (13.9)
**White**		27 (48.2)	3 (5.4)	7 (12.5)	9 (16.1)	10 (17.9)

[a]Other ethnic groups included: Tswana (n=1), Xhosa (n=1), Angolan (n=1), and unknown (n=2)

© 2023 Boucheron P et al. *JAMA Network Open.*

eFigure 3. Crude Kaplan-Meier Curves of OS After a BC Diagnosis in ABC-DO Women by Population Group and Macroregion

A)

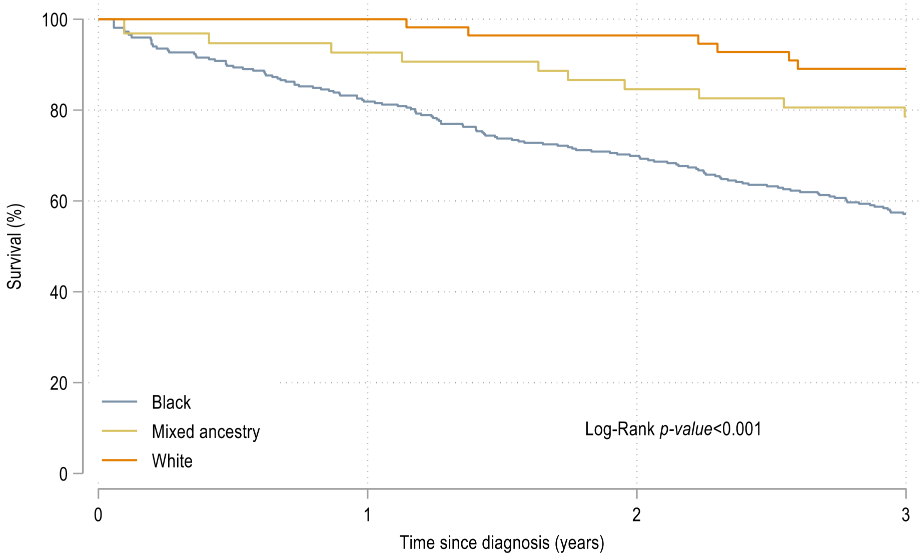

Population group	0	1	2	3
Black	300	247	220	178
Mixed ancestry	49	46	42	39
White	56	54	53	48

B)

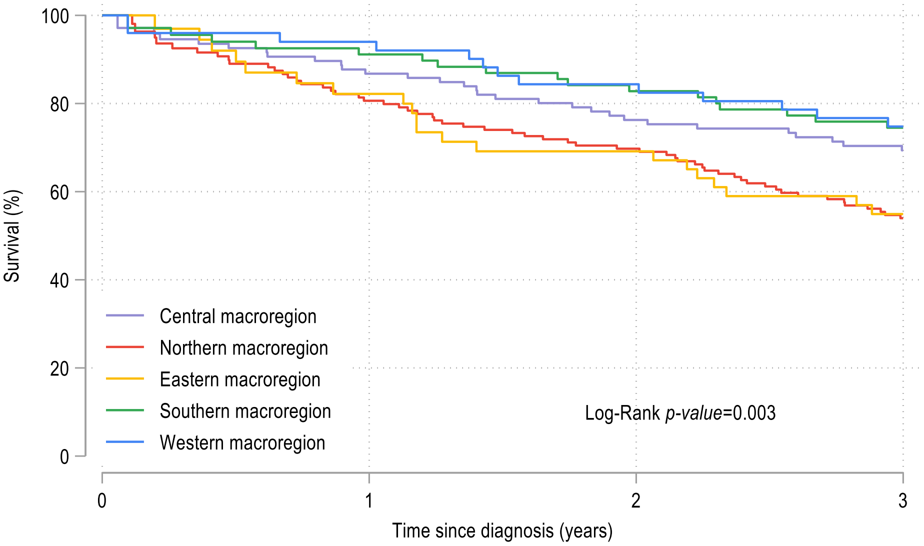

Macroregion of residence	0	1	2	3
Central	102	90	79	70
Northern	134	108	98	75
Eastern	47	36	34	27
Southern	71	65	60	54
Western	51	48	44	39

ABC-DO: African Breast Cancer – Disparities in Outcomes study; BC: breast cancer; OS: overall survival.
(A) Crude Kaplan-Meier curve of OS after a BC diagnosis in ABC-DO women, by population group (3-year overall mortality HRs $_{\text{age-HIV adjusted}}$ (95%CI): Black (1 (Ref)), Mixed ancestry (0.44 (0.23, 0.85)), White (0.23 (0.10, 0.52)); (B) Crude Kaplan-Meier curve of OS after a BC diagnosis in ABC-DO women, by macroregion of residence (3-year overall mortality HRs $_{\text{age-HIV adjusted}}$ (95%CI): Central region (1 (Ref)), Northern region (1.13 (0.70, 1.82)), Eastern region (1.54 (0.86, 2.75)), Southern region (0.75 (0.41, 1.38)), and Western region (0.67 (0.34, 1.31)).
Note: To avoid having sparse data, the 14 regions in the country were aggregated into five macroregions as follows: Western = Kunene and Erongo; Southern = Hardap and Karas.; Eastern = Omaheke and Otjozondjupa; Northern = Omusati, Oshana, Ohangwena, Oshikoto, Kavango West and Kavango East, and Zambezi; and Central = Khomas (the region where the country's capital, Windhoek, is located).

© 2023 Boucheron P et al. *JAMA Network Open.*

eFigure 4. Crude Kaplan-Meier OS Curves After BC Diagnosis in Black Women, by Ethnic Group and Macroregion

A)

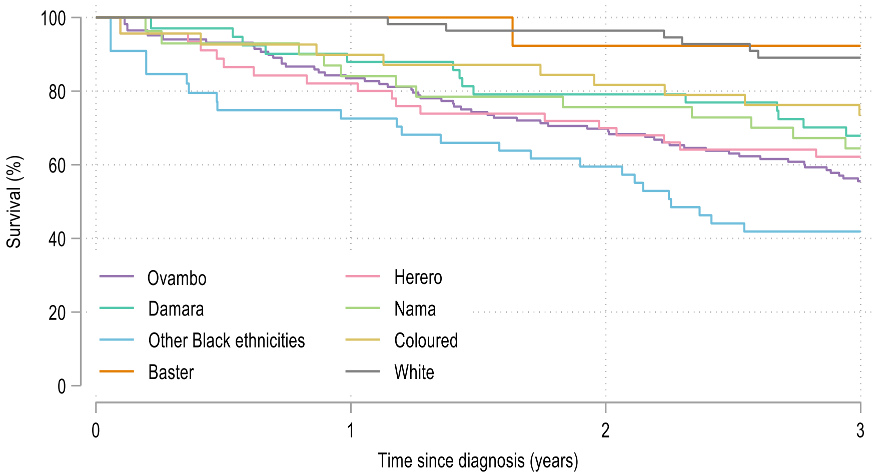

Ethnicity				
Ovambo	129	106	94	74
Herero	49	40	36	32
Damara	45	40	36	30
Nama	35	29	27	23
Other Black ethnicities	42	32	27	19
Coloured	36	33	30	27
Baster	13	13	12	12
White	56	54	53	48

B)

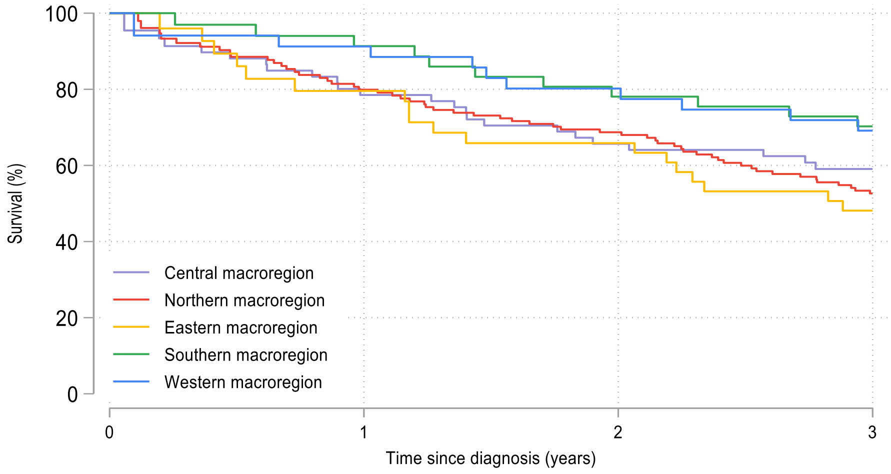

Macroregion of residence				
Central	60	49	41	35
Northern	130	104	94	72
Eastern	37	27	26	19
Southern	38	34	30	27
Western	35	33	29	25

Note: To avoid having sparse data, the 14 regions in the country were aggregated into five macroregions as follows: Western = Kunene and Erongo; Southern = Hardap and Karas.; Eastern = Omaheke and Otjozondjupa; Northern = Omusati, Oshana, Ohangwena, Oshikoto, Kavango West and Kavango East, and Zambezi; and Central = Khomas (the region where the country's capital, Windhoek, is located).
Note2: Fig.1A 3-year overall mortality HRs $_{\text{age-HIV adjusted}}$ (95%CI): Ovambo (4.86 (2.06, 11.45)), Herero (4.17 (1.64, 10.63)), Damara (3.34 (1.27, 8.75)), Nama (3.88 (1.45, 10.39)), Other Black ethnicities (6.23 (2.46, 15.80)), Coloured (2.62 (0.93, 7.39)), Baster (0.65 (0.08, 5.45)) and White (1 (Ref)).
Note3: Fig. 1B 3-year overall mortality HRs $_{\text{age-HIV adjusted}}$ (95%CI): Central macroregion (1 (Ref)), Northern macroregion (1.10 (0.67, 1.81)), Eastern macroregion (1.50 (0.80, 2.79)), Southern macroregion (0.69 (0.33, 1.41)), and Western macroregion (0.64 (0.30, 1.34)).

© 2023 Boucheron P et al. *JAMA Network Open.*

eFigure 5. Breast Cancer Journey in Women Included in ABC-DO in Namibia

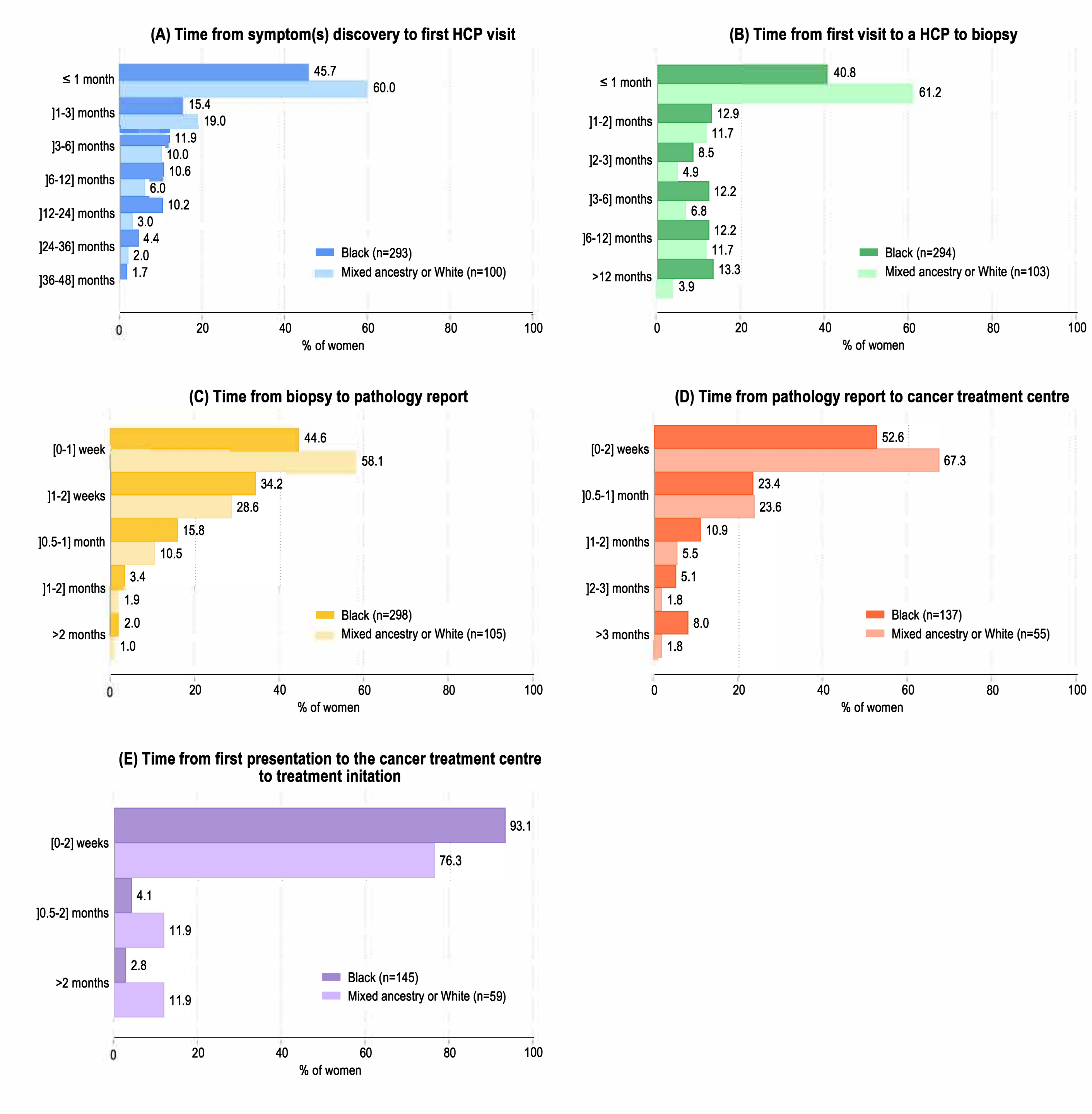

N.B: Panel figures (D) and (E) were restricted to non-metastatic women who were alive six months after the diagnosis and who initiated treatment at Windhoek Central Hospital.

© 2023 Boucheron P et al. *JAMA Network Open.*

eTable 4. Characteristics of Women With Long vs Shorter Precontact Interval in Namibia in ABC-DO[a]

		Pre-contact interval			
		≤90 days (n=258)	>90 days (n=135)	p-value[b]	p-value[c]
		No. (row%)	No. (row%)		
**Sociodemographic characteristics**					
Population group	Black	179 (61.1)	114 (38.9)	0.01	0.74
	Mixed ancestry	39 (79.6)	10 (20.4)		
	White	40 (78.4)	11 (21.6)		
Age at diagnosis (in years)	<40	43 (59.7)	29 (40.3)	0.75	0.86
	40-49	70 (70.0)	30 (30.0)		
	50-59	64 (66.0)	33 (34.0)		
	60-69	46 (66.7)	23 (33.3)		
	70+	35 (63.6)	20 (36.4)		
Macroregion of residence	Central	72 (72.7)	27 (27.3)	0.88	-
	Northern	78 (60.5)	51 (39.5)		
	Eastern	30 (63.8)	17 (36.2)		
	Southern	47 (68.1)	22 (31.9)		
	Western	31 (63.3)	18 (36.7)		
Education	None/Primary school	103 (56.3)	80 (43.7)	<0.001	0.01
	Secondary/high school	94 (67.6)	45 (32.4)		
	Technical/University	61 (85.9)	10 (14.1)		
Live with a partner	Yes	102 (70.8)	42 (29.2)	0.48	-
	No	156 (62.7)	93 (37.3)		
No. children at home	Median No. (IQR)	2 (0, 3)	2 (0, 4)	0.92	-
Young child at home	Yes (i.e., ≤5 years)	34 (60.7)	22 (39.3)	0.81	
	No	177 (66.0)	91 (34.0)		
Residential area	Urban	170 (70.0)	73 (30.0)	0.27	-
	Rural	88 (58.7)	62 (41.3)		
**Comorbidities**					
HIV status	Positive	31 (60.8)	20 (39.2)	0.64	-
	Negative/Unknown	227 (66.4)	115 (33.6)		
Comorbidities	Yes	147 (71.7)	58 (28.3)	0.02	0.051
	No	111 (59.0)	77 (41.0)		
**BC awareness**					
Heard about BC	Yes	238 (68.0)	112 (32.0)	0.04	-
	No/Don't know	20 (46.5)	23 (53.5)		

© 2023 Boucheron P et al. *JAMA Network Open.*

		Pre-contact interval			
		≤90 days (n=258) No. (row%)	>90 days (n=135) No. (row%)	p-value[b]	p-value[c]
Believed BC is potentially curable	Yes	220 (70.3)	93 (29.7)	0.004	-
	No/Don't know	38 (47.5)	42 (52.5)		
Interpretation of first symptom as a possible BC	Yes	58 (85.3)	10 (14.7)	0.001	0.002
	No	200 (61.5)	125 (38.5)		
**Barriers to access first visit to a HCP**					
Travel time from home to first HCP visit (in minutes)	≤60 minutes	218 (67.9)	103 (32.1)	0.18	-
	>60 minutes	40 (55.6)	32 (44.4)		
Difficulty to access healthcare	Yes	40 (51.3)	38 (48.7)	0.04	-
	No	218 (69.2)	97 (30.8)		
Belief barrier	Yes	4 (19.0)	17 (81.0)	<0.001	-
	No	254 (68.3)	118 (31.7)		
Lack of time	Yes	2 (16.7)	10 (83.3)	0.004	-
	No	256 (67.2)	125 (32.8)		
Husband	Yes	0 (0.0)	1 (100)	N/A	-
	No	258 (65.8)	134 (34.2)		
At least one barrier	Yes	49 (45.0)	60 (55.0)	<0.001	0.002
	No	209 (73.6)	75 (26.4)		
**BC characteristics**					
TNM stage at diagnosis[d]	Stage I	29 (11.2)	5 (3.7)	-	-
	Stage II	106 (41.1)	43 (31.9)		
	Stage III	100 (38.8)	58 (43.0)		
	Stage IV	23 (8.9)	29 (21.5)		

BC: Breast cancer; HCP: Healthcare practitioner; IQR: Inter-quartile range
[a]12 women with incorrect dates of first noticing symptoms (n=9) or incorrect dates of first visit to a HCP (n=6) were excluded
[b]P-values obtained from Wald tests from logistic regression models adjusted for population group (Black, Mixed ancestry and White women), and age at baseline (<40 years/40-49/50-59/60-69/70+ years)
[c]P-values obtained from Wald tests from logistic regression models adjusted for population group (Black, Mixed ancestry and White women), age at baseline (<40 years/40-49/50-59/60-69/70+ years), educational level (as continuous), interpretation of first symptom as possible BC (Yes/no), and self-report of at least one barrier to access the first visit to a HCP (Yes/no). To avoid multicollinearity, only the strongest determinant of BC awareness and of barriers to access first visit were included in the model.
[d]Not considered as a potential determinant of pre-contact interval

© 2023 Boucheron P et al. *JAMA Network Open.*

eTable 5. Characteristics of Women With Long vs Shorter Diagnostic Interval in Namibia in ABC-DO[a]

		Diagnostic interval			
		≤60 days (n=258)	>60 days (n=141)	p-value[b]	p-value[c]
		No. (row %)	No. (row %)		
**Sociodemographic characteristics**					
Population group	Black	178 (60.1)	118 (39.9)	0.01	0.01
	Mixed ancestry	35 (71.4)	14 (28.6)		
	White	45 (83.3)	9 (16.7)		
Age at diagnosis (in years)	<40	45 (61.6)	28 (38.4)	0.66	0.69
	40-49	65 (62.5)	39 (37.5)		
	50-59	62 (63.3)	36 (36.7)		
	60-69	51 (73.9)	18 (26.1)		
	70+	35 (63.6)	20 (36.4)		
Macroregion of residence	Central	71 (69.6)	31 (30.4)	0.77	-
	Northern	81 (62.3)	49 (37.7)		
	Eastern	27 (57.4)	20 (42.6)		
	Southern	48 (68.6)	22 (31.4)		
	Western	31 (62.0)	19 (38.0)		
Education	None/Primary school	116 (63.0)	68 (37.0)	0.95	-
	Secondary/high school	89 (62.7)	53 (37.3)		
	Technical/University	53 (72.6)	20 (27.4)		
Live with a partner	Yes	110 (73.8)	39 (26.2)	0.02	0.06
	No	148 (59.2)	102 (40.8)		
No. children at home	Median No. (IQR)	2 (0, 4)	2 (0, 3)	0.67	-
Young child at home	Yes (i.e., ≤5 years)	38 (66.7)	19 (33.3)	0.28	-
	No	184 (67.6)	88 (32.4)		
Residential area	Urban	162 (65.3)	86 (34.7)	0.40	-
	Rural	96 (63.6)	55 (36.4)		
**Comorbidities**					
HIV status	Positive	33 (63.5)	19 (36.5)	0.61	-
	Negative/Unknown	225 (64.8)	122 (35.2)		
Comorbidities	Yes	134 (64.4)	74 (35.6)	0.46	-
	No	124 (64.9)	67 (35.1)		
**Pre-contact journey**					

© 2023 Boucheron P et al. *JAMA Network Open.*

		Diagnostic interval			
		≤60 days (n=258)	>60 days (n=141)	p-value[b]	p-value[c]
		No. (row %)	No. (row %)		
Long pre-contact interval	Yes (i.e., >90 days)	103 (76.3)	32 (23.7)	<0.001	0.001
	No	150 (58.1)	108 (41.9)		
**Diagnostic journey**					
After the first visit, ≥1 long travel to reach a HCP visit (>60 minutes)[d]	Yes	92 (55.1)	75 (44.9)	0.26	-
	No	102 (62.2)	62 (37.8)		
After the first visit, ≥1 barrier to reach a HCP visit[d,e]	Yes	40 (40.0)	60 (60.0)	<0.001	0.001
	No	154 (66.7)	77 (33.3)		
**BC characteristics**					
TNM stage at diagnosis[f]	Stage I	29 (11.2)	5 (3.7)	-	-
	Stage II	106 (41.1)	43 (31.9)		
	Stage III	100 (38.8)	58 (43.0)		
	Stage IV	23 (8.9)	29 (21.5)		

BC: Breast cancer; HCP: Healthcare practitioner; IQR: Inter-quartile range
[a]6 women with incorrect dates of first visit to a HCP were excluded
[b]P-values obtained from Wald tests from logistic regression models adjusted for population group (Black, Mixed ancestry and White women), and age at baseline (<40 years/40-49/50-59/60-69/70+ years)
[c]P-values obtained from Wald tests from logistic regression models adjusted for population group (Black, Mixed ancestry and White women), age at baseline (<40 years/40-49/50-59/60-69/70+ years), living with a partner (Yes/no), pre-contact interval (≤ / > 90 days), and encountering at least one barrier to reach a HCP after the first contact with the healthcare system (Yes/No).
[d]P-values reported for Yes v No (68 women had only one HCP visit before their BC diagnosis, including 64 women with a diagnostic interval ≤60 days, and 4 with a diagnostic interval >60 days)
[e]Mainly represented by difficulties to access transportation (not available, cost too high), or to obtain an appointment
[f]Not considered as potential determinant of diagnostic interval

© 2023 Boucheron P et al. *JAMA Network Open.*

eFigure 6. Geographical Barriers to Access Health Care in Namibia in ABC-DO

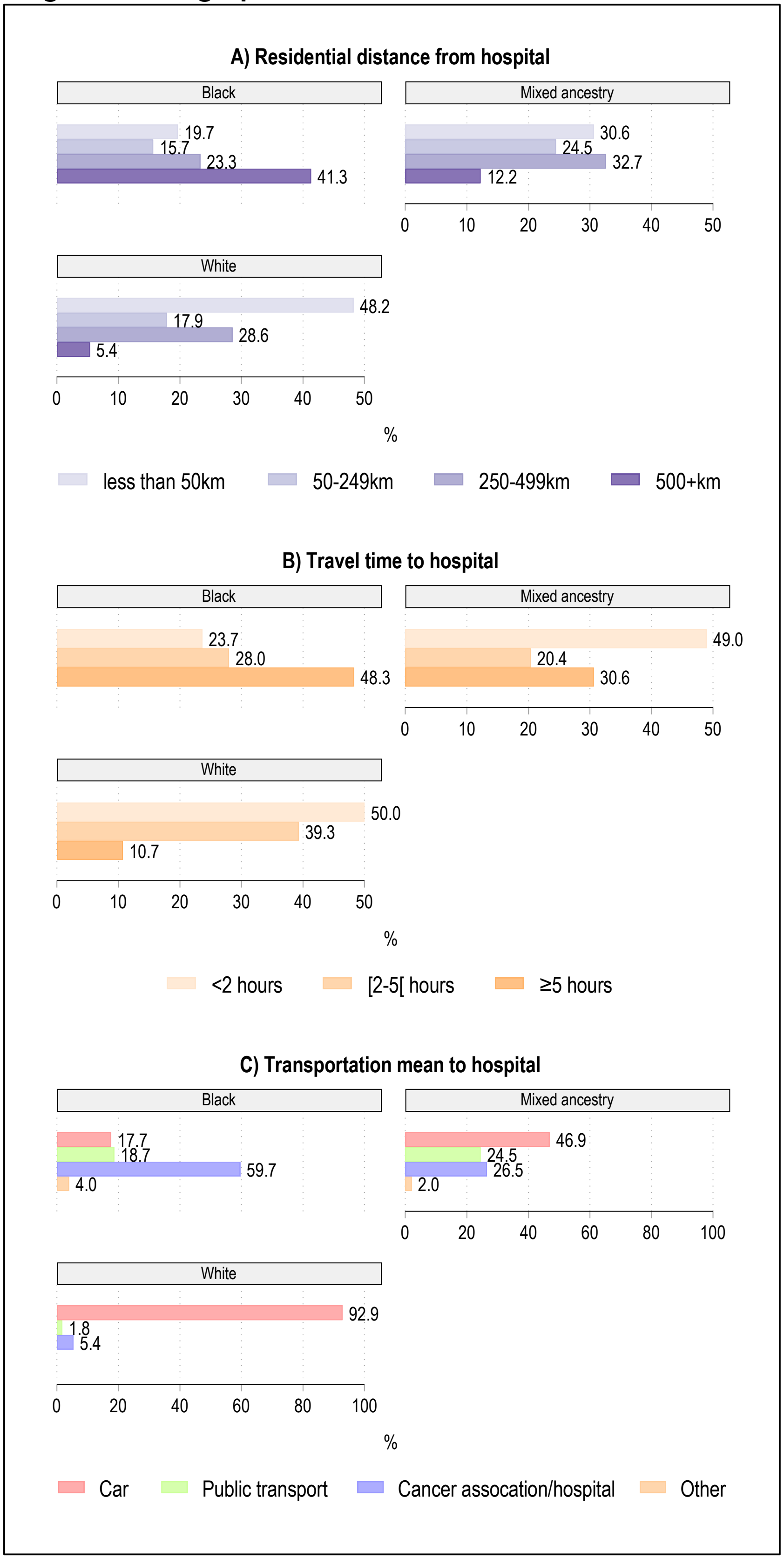

© 2023 Boucheron P et al. *JAMA Network Open.*

eTable 6. Characteristics of Women With Long vs Shorter Treatment Interval From Diagnosis in Namibia in ABC-DO[a]

		Treatment interval			
		≤30 days (n=155)	>30 days (n=177)	p-value[b]	p-value[c]
		No. (row %)	No. (row %)		
**Sociodemographic characteristics**					
Population group	Black	105 (44.7)	130 (55.3)	0.54	0.74
	Mixed ancestry	24 (53.3)	21 (46.7)		
	White	26 (50.0)	26 (50.0)		
Age at diagnosis (in years)	<40	28 (46.7)	32 (53.3)	0.54	0.30
	40-49	39 (42.4)	53 (57.6)		
	50-59	37 (45.1)	45 (54.9)		
	60-69	30 (57.7)	22 (42.3)		
	70+	21 (45.7)	25 (54.3)		
Macroregion of residence	Central	44 (53.0)	39 (47.0)	0.67	-
	Northern	42 (40.0)	63 (60.0)		
	Eastern	16 (44.4)	20 (55.6)		
	Southern	31 (50.0)	31 (50.0)		
	Western	22 (47.8)	24 (52.2)		
Education	None/Primary school	64 (44.8)	79 (55.2)	0.04	0.08
	Secondary/high school	49 (41.2)	70 (58.8)		
	Technical/University	42 (60.0)	28 (40.0)		
Live with a partner	Yes	64 (48.5)	68 (51.5)	0.57	-
	No	91 (45.5)	109 (54.5)		
No. children at home	Median No. (IQR)	2 (0, 3)	2 (0, 4)	0.15	-
Young child at home	Yes (i.e., ≤5 years)	19 (43.2)	25 (56.8)	0.79	-
	No	110 (48.7)	116 (51.3)		
Residential area	Urban	103 (47.5)	114 (52.5)	0.96	-
	Rural	52 (45.2)	63 (54.8)		
**Comorbidities**					
HIV status	Positive	14 (33.3)	28 (66.7)	0.11	-
	Negative/Unknown	141 (48.6)	149 (51.4)		
Other comorbidities	Yes	79 (46.7)	90 (53.3)	0.73	-
	No	76 (46.6)	87 (53.4)		
**Pre-contact journey**					
Long pre-contact interval	Yes (i.e., >90 days)	45 (46.4)	52 (53.6)	>0.99	-
	No	106 (47.1)	119 (52.9)		
**Diagnostic journey**					

© 2023 Boucheron P et al. *JAMA Network Open.*

		Treatment interval			
		≤30 days (n=155)	>30 days (n=177)	p-value[b]	p-value[c]
		No. (row %)	No. (row %)		
Long diagnostic interval	Yes (i.e., >60 days)	57 (46.0)	67 (54.0)	0.98	-
	No	97 (47.5)	107 (52.5)		
**BC characteristics**					
TNM stage at diagnosis	Stage I	18 (50.0)	18 (50.0)	0.90	-
	Stage II	67 (45.9)	79 (54.1)		
	Stage III	70 (46.7)	80 (53.3)		
**Barriers to access the oncology centre (i.e., WCH)**					
Distance from home to WCH (in Km)	<50km	44 (53.0)	39 (47.0)	0.61	-
	50-249	27 (49.1)	28 (50.9)		
	250-499	40 (44.4)	50 (55.6)		
	≥500	44 (42.3)	60 (57.7)		
Travel time from home to WCH (in hours)	<2	53 (52.5)	48 (47.5)	0.14	-
	[2-5[	48 (48.0)	52 (52.0)		
	≥5	54 (41.2)	77 (58.8)		
Transportation mean from home to WCH	Car	62 (56.4)	48 (43.6)	<0.001	-
	Cancer association/hospital	54 (35.3)	99 (64.7)		
	Public transport/Other[d]	39 (56.5)	30 (43.5)		
≥1 barrier to access WCH	Yes	12 (27.3)	32 (72.7)	0.01	0.02
	No/Unknown[e]	143 (49.7)	145 (50.3)		

BC: Breast cancer; IQR: inter-quartile range; WCH: Windhoek Central Hospital
[a]Among 332 non-metastatic treated women with known treatment interval (i.e., from BC diagnosis to treatment initiation)
[b]P-values obtained from Wald tests from logistic regression models adjusted for population group (Black, Mixed ancestry and White women), and age at baseline (<40 years/40-49/50-59/60-69/70+ years)
[c]P-values obtained from Wald tests from logistic regression models adjusted for population group (Black, Mixed ancestry and White women), age at baseline (<40 years/40-49/50-59/60-69/70+ years), educational level (as continuous), and encountering at least one barrier to access the cancer treatment center (yes/no or unknown). To avoid multicollinearity, only the strongest determinant of barriers to access the oncology center was included into the model.
[d]9 Black women took other transportation mean to reach the hospital
[e]Barriers to access the hospital were unknown for 8 Black women

© 2023 Boucheron P et al. *JAMA Network Open.*

eTable 7. Sensitivity Analysis Regarding Recommended Multimodal Treatment (Surgery Plus Chemotherapy) Completion in Namibia in ABC-DO[a]

			Black	Mixed ancestry	White
			**No. (%)**	**No. (%)**	**No. (%)**
**Among women for whom MT was indicated, irrespective of whether it was received**			**n=236**	**n=39**	**n=38**
MT completed (GBCI pillar III KPI)[b]	Yes	MT initiated and chemotherapy completed	112 (47.5)	22 (56.4)	23 (60.5)
		MT timely initiated[c]	57 (24.2)	15 (38.5)	13 (34.2)
		MT initiation delayed[c]	55 (23.3)	7 (17.9)	10 (26.3)
	No	MT initiated but chemotherapy not completed	37 (15.7)	7 (17.9)	5 (13.2)
		Chemotherapy ended before completion	33 (14.0)	6 (15.4)	4 (10.5)
		Woman died within 6 months of chemotherapy initiation	0 (0.0)	0 (0.0)	0 (0.0)
		Chemotherapy completion unknown	4 (1.7)	1 (2.6)	1 (2.6)
		MT not initiated	87 (36.9)	10 (25.6)	10 (26.3)
		Chemotherapy not initiated	26 (11.0)	2 (5.1)	7 (18.4)
		Surgery not received	37 (15.7)	5 (12.8)	2 (5.3)
		No surgery nor chemotherapy	24 (10.2)	3 (7.7)	1 (2.6)
**Among women with negative or unknown HIV status for whom MT was indicated and surgery and/or chemotherapy received**			**n=178**	**n=33**	**n=37**
MT completed (GBCI pillar III KPI)[b]	Yes	MT initiated and chemotherapy completed	93 (52.2)	21 (63.6)	23 (62.2)
		MT timely initiated[c]	50 (28.1)	15 (45.5)	13 (35.1)
		MT initiation delayed[c]	43 (24.2)	6 (18.2)	10 (27.0)
	No	MT initiated but chemotherapy not completed	30 (16.9)	5 (15.2)	5 (13.5)
		Chemotherapy ended before completion	26 (14.6)	4 (12.1)	4 (10.8)
		Woman died within 6 months of chemotherapy initiation	0 (0.0)	0 (0.0)	0 (0.0)

© 2023 Boucheron P et al. *JAMA Network Open.*

			Black	Mixed ancestry	White
			No. (%)	No. (%)	No. (%)
		Chemotherapy completion unknown	4 (2.2)	1 (3.0)	1 (2.7)
		MT not initiated	55 (30.9)	7 (21.2)	9 (24.3)
		Chemotherapy not initiated	25 (14.0)	2 (6.1)	7 (18.9)
		Surgery not received	30 (16.9)	5 (15.2)	2 (5.4)
**Among women aged ≤75 years for whom MT was indicated and surgery and/or chemotherapy received**			**n=200**	**n=33**	**n=33**
MT completed (GBCI pillar III KPI)[b]	Yes	MT initiated and chemotherapy completed	112 (56.0)	22 (66.7)	23 (69.7)
		MT timely initiated[c]	57 (28.5)	15 (45.5)	13 (39.4)
		MT initiation delayed[c]	55 (27.5)	7 (21.2)	10 (30.3)
	No	MT initiated but chemotherapy not completed	36 (18.0)	7 (21.2)	4 (12.1)
		Chemotherapy ended before completion	33 (16.5)	6 (18.2)	4 (12.1)
		Woman died within 6 months of chemotherapy initiation	0 (0.0)	0 (0.0)	0 (0.0)
		Chemotherapy completion unknown	3 (1.5)	1 (3.0)	0 (0.0)
		MT not initiated	52 (26.0)	4 (12.1)	6 (18.2)
		Chemotherapy not initiated	19 (9.5)	0 (0.0)	4 (12.1)
		Surgery not received	33 (16.5)	4 (12.1)	2 (6.1)

BC: Breast cancer; GBCI: Global Breast Cancer Initiative; KPI: Key Performance Indicator; MT: multimodal treatment
[a]All analyses were performed in non-metastatic women who were still alive six months after BC diagnosis
[b]>85% of the total cumulative chemotherapy completed defined as ≥5 cycles of fluorouracil, doxorubicin, and cyclophosphamide therapy (or an equivalent regimen) administered within 15 weeks of chemotherapy initiation, or ≥7 cycles of fluorouracil, doxorubicin, cyclophosphamide, and taxane therapy within 28 weeks, after the first dose or cycle (the recommended timeframe is within 24 weeks of diagnosis
[c]Treatment considered timely initiated if surgery was performed or chemotherapy was initiated within 30 days of BC diagnosis

© 2023 Boucheron P et al. *JAMA Network Open.*

eReferences

1. Pheby D, Roumagnac M, Registry TC, Albi F. European Network of Cancer Registries (ENCR) Recommendations for coding Incidence Date.

© 2023 Boucheron P et al. *JAMA Network Open.*
