# Supplementary material for: Analysis of the Breast Cancer Journey in Namibia
Source: JAMA Netw Open. 2023 Nov 3;6(11):e2341402. doi: 10.1001/jamanetworkopen.2023.41402 (PMC10625043; doi:10.1001/jamanetworkopen.2023.41402)
Supplement: Supplement 2. — Data Sharing Statement [file jamanetwopen-e2341402-s002.pdf]

Data Sharing Statement

Boucheron. Analysis of the Breast Cancer Journey in Namibia. *JAMA Netw Open*. Published November 03, 2023. doi:10.1001/jamanetworkopen.2023.41402

Data
Data available: No

Additional Information
Explanation for why data not available: All data generated or analysed during this study are included in this published article and its supplementary information files. Further information is available from the corresponding author upon request.
